# Real-Time Imaging of Plasmonic Concentric Circular Gratings Fabricated by Lens–Axicon Laser Interference Lithography

**DOI:** 10.3390/mi14111981

**Published:** 2023-10-26

**Authors:** Mahyar Mazloumi, Ribal Georges Sabat

**Affiliations:** Department of Physics and Space Science, Royal Military College of Canada, P.O. Box 17000, STN Forces, Kingston, ON K7K 7B4, Canada; georges.sabat@rmc-cmr.ca

**Keywords:** concentric circular grating, laser interference lithography, azobenzene, plasmonics, imaging

## Abstract

Concentric circular gratings are diffractive optical elements useful for polarization-independent applications in photonics and plasmonics. They are usually fabricated using a low-throughput and expensive electron beam lithography technique. In this paper, concentric circular gratings with selectable pitch values were successfully manufactured on thin films of azobenzene molecular glass using a novel laser interference lithography technique utilizing Bessel beams generated by a combined lens–axicon configuration. This innovative approach offers enhanced scalability and a simplified manufacturing process on larger surface areas compared to the previously reported techniques. Furthermore, the plasmonic characteristics of these concentric circular gratings were investigated using conventional spectrometric techniques after transferring the nanostructured patterns from azobenzene to transparent gold/epoxy thin films. In addition, the real-time imaging of surface plasmon resonance colors transmitted from the concentric circular gratings was obtained using a 45-megapixel digital camera. The results demonstrated a strong correlation between the real-time photographic technique and the spectroscopy measurements, validating the efficacy and accuracy of this approach for the colorimetric studying of surface plasmon resonance responses in thin film photonics.

## 1. Introduction

A variety of nanofabrication techniques have been reported thus far in the literature for the fabrication of diverse micro- and nanostructures on thin films made with photoactive azobenzene materials [1]. Among these techniques, laser interference lithography has been predominantly utilized due to the well-documented photomechanical behavior of azobenzene materials, in which a macroscopic molecular mass movement results in the formation of surface relief patterns on the azobenzene film [2].

By manipulating the laser interference pattern, a wide array of in-plane diffractive optical elements can be produced on azobenzene materials, including linear surface relief gratings (SRG) [3], crossed surface relief gratings (CSRG) [4], concentric circular gratings [5], and metasurfaces [6]. These elements can exhibit constant or chirped pitch values spanning from the ultraviolet range to the millimeter scale [7,8,9].

Concentric circular gratings stand out as particularly intriguing due to their unique radial surface features, which make them highly desirable in applications such as alignment marks in lithography techniques [10], distributed feedback lasers [11,12], light-trapping elements in luminescent enhancing applications [13,14,15], plasmonic solar cells [16,17] and for the generation of radially and azimuthally polarized light using plasmonics [18,19].

Previous theoretical research has demonstrated that the optimized intensity of surface plasmon resonance excited on concentric circular gratings can surpass that of linear gratings by over an order of magnitude [17]. This breakthrough has the potential to experimentally revolutionize various fields reliant on plasmonic enhancement and light entrapment, including the photovoltaic industry. In addition, concentric circular gratings are good candidates for the generation of radially polarized light, which is crucial in many applications such as laser micromachining, as it yields sharper focus and plasmonic enhancement [19,20]. Consequently, it becomes imperative to develop cost-effective and scalable methods for fabricating concentric optical elements, such as employing large-area optical lithography on azobenzene materials in place of more expensive low-throughput electron beam lithography reported elsewhere [11,21].

Recent advancements in light interference lithography techniques have revealed that not only Gaussian laser beams can be harnessed to create various optical elements, but also that Bessel laser beams proved to be excellent candidates, particularly for fabricating concentric optical metasurfaces in azobenzene materials [5]. Bessel beams are a class of non-diffracting optical beams that have a ring-shaped intensity profile maintained over a significant distance. This property makes them useful in applications where a long and nearly undistorted propagation is required [22,23]. One way to generate Bessel beams is using axicons [24]. A single Bessel laser beam can be generated by an axicon and employed to fabricate fixed-pitch concentric circular gratings with a pitch value related to the base angle of the axicon [5]. One limitation of this approach is that the axicon geometry, with its fixed base angle, can only produce a circular grating with a singular fixed pitch value. This constraint hinders scalability and increases manufacturing costs, since different axicon geometries are needed to create various pitch concentric circular gratings. 

Surface plasmon resonance (SPR) is a phenomenon in which a collective oscillation of free electrons occurs at the interface between a dielectric medium and a metal surface. SPR is highly sensitive to changes in the refractive index of the adjacent medium, which makes it a valuable tool for spectral-based sensing applications. SPR imaging (SPRi) is an analytical technique that allows for the real-time label-free visualization and characterization of nanoscale interactions that occur either locally on the surface of plasmonic nanoparticles or on a larger scale on the surface of nanostructured substrates [25,26,27]. 

SPRi techniques presented thus far in the literature are mainly focused on molecular-level sensing using microscopes [27]. However, as the technology improves, humans are becoming more dependent on their smartphones, and they can use their cameras in real-time label-free photocolorimetric sensing and detections [28]. Therefore, it is vital to develop techniques that can use regular digital cameras in the colorimetric detection of surface plasmon resonances. 

In this paper, a novel technique is introduced to fabricate concentric circular gratings with different pitch values in thin films of azobenzene molecular glass (gDR1) using a combined lens–axicon geometry. Furthermore, the plasmonic behavior of these concentric circular gratings is analyzed using conventional spectrometric techniques, in addition to real-time imaging using a high-resolution digital camera. This study paves the way for the development of new plasmonic devices with applications in real-time imaging and colorimetric sensing.

## 2. Materials and Methods

**Figure 1:**
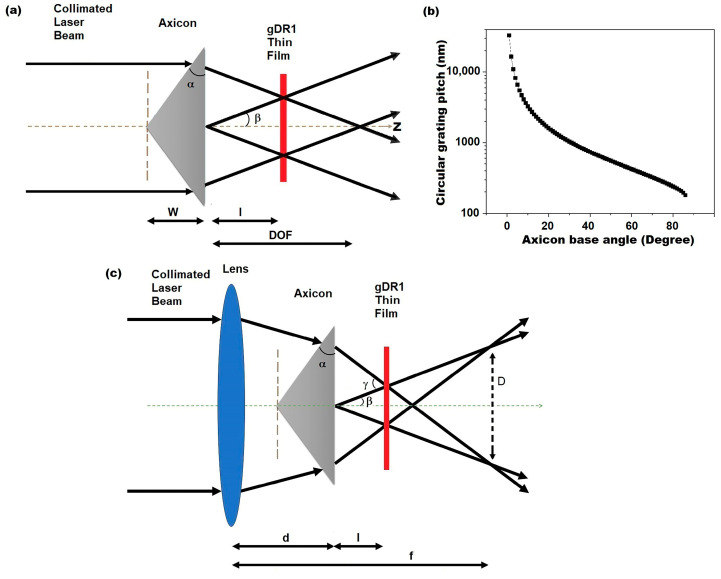
(**a**) Schematics of axicon interference lithography; (**b**) dependence of the circular grating pitch value to the axicon base angle in axicon interference lithography; (**c**) schematics of the lens–axicon interference lithography.

Disperse Red-1 azobenzene molecular glass (gDR1) powder was synthesized via a process described previously [29]. A solution of 3 wt% of gDR1 was dissolved in Dichloromethane (CH_2_Cl_2_), filtered using a 0.45 μm syringe filter and then spin-coated at 1000 RPM on clean Corning 0215 soda lime microscope slides (38 × 38 × 1 mm^3^). The gDR1-coated slides were dried at 80 °C in air for 15 min to remove any possible trapped solvents. Optically uniform films of gDR1 were obtained with a thickness of 300 nm, as measured using a DektakXT stylus surface profiler (Bruker, San Jose, CA, USA). Several tiny scratches were made on different spots of the gDR1 film for this purpose. The surface profilometer uses a diamond stylus with controlled vertical and lateral movements to measure small deviations in the stylus position, providing a direct assessment of the surface elevation or film thickness where the scratches are created. A continuous wave (CW) Gaussian laser beam from a diode-pumped solid-state laser (Coherent, Verdi V6, 532 nm) was passed through a spatial filter and made circularly polarized using a quarter-wave plate. The beam was expanded and collimated before being passed through a variable iris to control its size. Then, it was normally incident on a spherical convex lens with a focal length of *f*. At a distance *d* from the convex lens and before its focal point, an axicon (EKSMA Optics, Vilnius, Lithuania) with a base angle of *α* and thickness of *w* was placed along the laser path. The diameter of the axicon was approximately 25 mm. The converging laser beam passing through the convex lens and the axicon created an interference pattern on the gDR1 film that was positioned normally to the incident beam at a distance of *l*, as illustrated in Figure 1. The irradiance of the incident collimated laser beam on the convex lens was kept constant at 115 mW·cm^−2^ for all exposures, and the inscription time was set at 500 s. The inscription area of the concentric circular gratings was dependent on the diameter of the axicon, as well as the diameter of the incident laser beam. The maximum grating diameter fabricated in this study was approximately 10 mm. However, using a wider laser beam and/or a larger axicon, the grating area could be even bigger. After the laser inscription step, the morphology, modulation depths and pitch value of the resulting concentric circular gratings were measured using a Dimension Edge atomic force microscope (AFM, Bruker, San Jose, CA, USA) and analyzed using the Nanoscope analysis v1.5 software of the AFM. The surface topography and texture of the obtained nanostructures on the gDR1 film was assessed using a 3D ContourX-200 optical profilometer (Bruker, San Jose, CA, USA). A 5 mW He-Ne laser with a wavelength of 632.8 nm was used to confirm the resulting grating pitch by measuring the angles between the 0 and ±1 diffraction order (m) according to the grating equation (Λ = m*λ*/sin*θ*). Subsequently, a thin layer of gold (10 nm) was sputtered onto the gDR1 thin film using a plasma coater (Quorum, Laughton, UK). The gold-coated circular gratings were transferred onto a transparent epoxy thin film using a UV-nanoimprint lithography technique by sandwiching the epoxy in between a glass slide and the gold-coated circular grating. The epoxy was UV-cured for 2 min and then peeled off, preserving the shape of gratings and the gold coating. The surface plasmon resonance (SPR) spectra of the resulting concentric circular gratings were recorded in transmission in air using a spectrometer (Ocean Insight, Orlando, FL, USA). White light was made horizontally polarized and illuminated the gratings at normal incidence. A secondary vertical polarizer was placed in line with the optical path after the gold-coated circular grating to eliminate the background effect. To perform the real-time imaging, a Nikon D850 DSLR camera (Nikon Canada Inc., Mississauga, ON, Canada) with 45-megapixel resolution was used in the setup described above. The videos were taken in Full HD mode with 30 fps speed.

## 3. Results and Discussion

Once a collimated Gaussian beam with wavelength of *λ* is incident on an axicon with a base angle of *α* (Figure 1a), it can be shown through Snell’s Law and simple trigonometry that the angle between the interfering laser beams (*β*) is constant and only depends on the base angle (*α*) and the refractive index of the axicon (*n_a_*). The angle between the interfering inscribing laser beams (*β*) can be calculated as follows [5]:

(1) $$\beta =\sin^{-1}(n_{a}\sin\left(\alpha -\sin^{-1}(\frac{\sin\alpha }{n_{a}}\right)))$$

An axicon delivers a decent estimation of a virtually non-diffracting zero-order Bessel beam within its depth of focus (DOF) where the size and shape of the central spot is preserved [24]. Within DOF (Figure 1a), the interfering beams create a circular pattern with a constant pitch value (Λ) that can be calculated as follows:

(2) $$\Lambda =\frac{\lambda }{2\sin(\beta )}$$

Equations (1) and (2) are plotted in Figure 1b to illustrate how the pitch of the circular interference pattern varies with the base angle of the axicon (*α*). For *α* = 1, the circular pattern pitch value is approximately 30 μm, while for *α* > 30°, the pitch value of the concentric circular gratings falls below 1 μm, which is comparable to subwavelength nanostructures. The interference patterns of Bessel beams caused by an axicon can be inscribed on thin films of azobenzene molecular glass, as shown in our previous work [5]. However, the main drawback of this axicon interference lithography method is that each base angle (*α*) corresponds to only one pitch value. Therefore, to make concentric circular gratings with different pitch values, different axicons with specified base angles are required. To overcome this problem, a new approach is presented here. 

An axicon manufactured by EKSMA Optics with a base angle of *α* = 45.0 ± 0.5° was used in this study. Assuming that the wavelength of the inscription laser beam λ = 532 nm, and the refractive index of the axicon *n_a_* = 1.4607 at the inscription wavelength, the constant pitch value of the concentric circular grating that can be obtained by this axicon (using Equations (1) and (2)) is equal to Λ = 660 nm (sample C1). 

When a spherical convex lens is added to the setup before the axicon (Figure 1c), the angle between the interfering inscribing Bessel beams (*γ* in this case) will depend on the focal length of the convex lens (*f*), the distance between the lens and axicon (*d*) and the distance between the gDR1 thin film and the axicon (*l*). In this configuration, the laser beam creates a ring pattern with a diameter *D* at the focal point of the lens, as shown in Figure 1c and calculated below [30]:

(3) D=2β(f−d)

Here, *β* is obtained from Equation (1). Now, the angle between the interfering inscribing beams (*γ*) is:

(4) $$\gamma \approx \frac{D}{\left(f-d-l\right)}$$

and the pitch value of the concentric circular grating obtained through this lens–axicon configuration is:

(5) $$\Lambda =\frac{\lambda }{2\sin(\frac{\gamma }{2})}$$

Therefore, the concentric circular gratings with different pitch values can now be fabricated using the axial movement of the lens and the gDR1 thin film compared to an axicon (Figure 1c).

**Figure 2:**
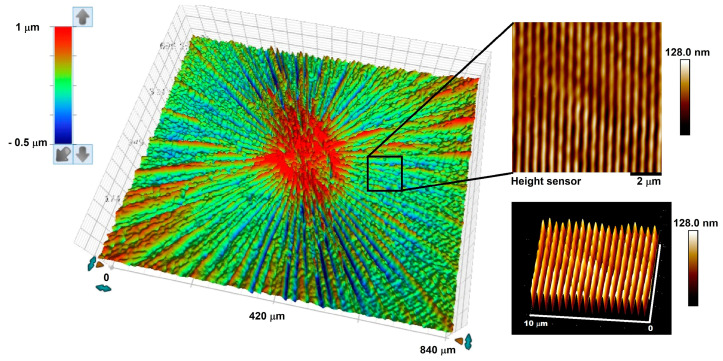
(**left**) 3D optical thin film topography of a typical concentric circular grating around its center and (**right**) corresponding 2D and 3D atomic force microscopy images of an area marked close to center.

Using this technique, five different concentric circular gratings were fabricated using the same axicon, which had a base angle of *α* = 45.0 ± 0.5°, on azobenzene molecular glass thin films with pitch values of 660 nm (C1), 584 nm (C2), 574 nm (C3), 553 nm (C4) and 469 nm (C5). Figure 2 shows the 3D optical profilometry image from the surface of a typical concentric circular grating thin film (sample C4). The optical profile shows a texture attributed to an area of 800 × 800 μm around the center of the circular grating. The topography of the film containing the radial grooves and central distortions on the surface of the gDR1 thin film can be clearly seen in this image. This is caused by the optical aberrations of the axicon and the high intensity of the Bessel beam around its center, which damaged the film [31]. However, when one focuses on areas beyond the central region, clear nanostructures, corresponding to constant-pitch circular gratings, are distinguished in radial positions. A zoomed-in 2D and 3D atomic force microscopy (AFM) imaging of a smaller area (10 × 10 μm) approximately 200 μm away from the center revealed the surface relief nanostructures as a portion of a concentric circular pattern (Figure 2). 

The plasmonic behavior of the fabricated concentric circular gratings was examined using spectroscopic and real-time camera imaging techniques. To eliminate any optical response related to the azobenzene film, all the fabricated concentric circular gratings were transferred from the gDR1 thin films onto transparent epoxy films using a UV-nanoimprint lithography technique. To facilitate this process and to preserve the transparency of the films, a 10 nm gold film was deposited onto the gDR1 films prior to the transfer. 

Based on theory [27], SPR occurs at the interface between a dielectric medium and a nanostructured thin metal layer when the incident light polarization is along the linear grating vector (or perpendicular to the grating lines). Consequently, in a gold-coated concentric circular grating, any linearly polarized light is along the grating vector and should excite SPR, according to the following equation:

(6) $$\lambda_{SP}=\Lambda n_{d}\left(\sqrt{\frac{\varepsilon_{r,m}^{’}}{n_{d}^{2}+\varepsilon_{r,m}^{’}}}\pm \sin\theta_{i}\right)$$

where Λ is the pitch value of the concentric circular grating structure, *n_d_* is the refractive index of the dielectric medium (air in this case), $\varepsilon_{r,m}^{’}$ is the real part of the permittivity of the metallic film (i.e., gold) and *θ_i_* is the light incidence angle (*θ_i_* = 0 at normal incidence). 

**Figure 3:**
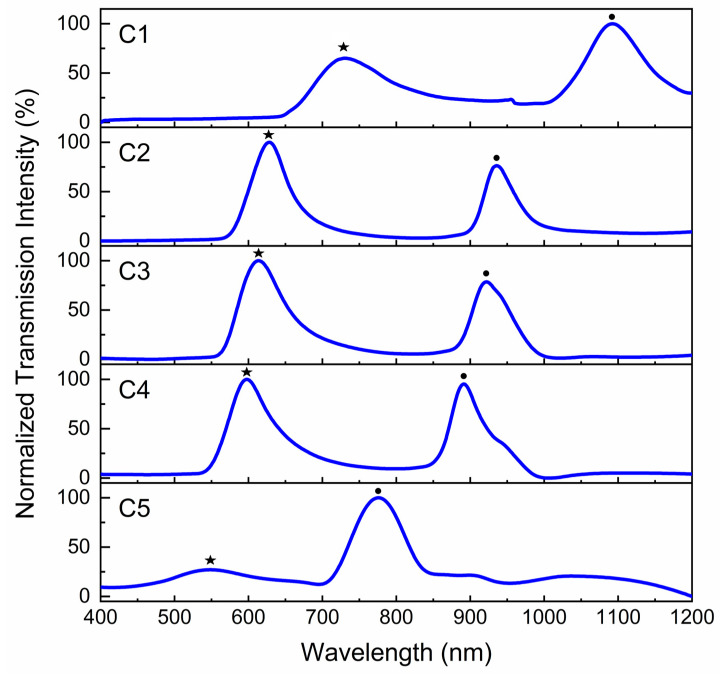
Normalized surface plasmon resonance (SPR) in transmission through the gold-coated concentric circular gratings on epoxy substrate excited by normally incident light in air. The concentric circular gratings were placed in between crossed polarizers. SPR peaks at air/gold interface are marked by stars and SPR peaks at gold/epoxy interface are marked by dots.

The SPR spectra of the gold-coated concentric circular grating samples noted as C1 to C5 on epoxy film were measured in transmission, with air being the dielectric medium over the gratings, and using two different Ocean optics spectrometers in the visible and NIR regions (Figure 3). The light from a Halogen lamp was horizontally polarized and normally incident to the circular grating surface. Then, the transmitted light from the concentric circular gratings passed through a secondary polarizer perpendicular to the first one and was collected by the collimating lens connected to the spectrometer. In this configuration of the crossed polarizers, the background white light is eliminated before reaching the spectrometer. However, only light that passed through the SPR energy conversion process is transmitted in a narrow band [26].

The plots in Figure 3 are the normalized transmission through the concentric circular gratings. Generally, surface plasmon resonance is observed as positive peaks in transmission and negative dips in reflection at the wavelength calculated according to Equation (6). However, once a plasmonic device is placed in between crossed polarizers, one should always detect a positive peak in both transmission and reflection. Here, the SPR plots of each concentric circular grating in Figure 3 are composed of double positive peaks marked by a star and a dot, as summarized in Table 1. 

The first peaks identified with stars in Figure 3 and in Table 1 correspond to the SPR wavelengths being excited on the concentric circular gratings according to Equation (6) at the interface between air and gold. The second peaks at higher wavelengths, identified with dots in Figure 3, are associated with the SPR being excited at the interface between gold and the epoxy film due to the higher refractive index of epoxy compared to air. For sample C5, the peak at 545 nm is almost eliminated because the SPR is absorbed by the gold thin film (Figure 3).

The concentric circular gratings in this study were placed in between crossed polarizers and in such a configuration that no light would pass through to the spectrometer except the surface plasmon resonance, as previously explained. Incident horizontally polarized light on the concentric circular gratings excites SPR at the air/gold and gold/epoxy interfaces, and this signal is transmitted as radially polarized light due to the polarization conversion phenomenon which occurs at the circular grating, and, therefore, the light could pass through the second polarizer and reach the detector. The SPR peaks excited at the air/gold interface (first peaks in Figure 3) are within the visible range and therefore are the focus of the imaging studies in this paper. 

**Figure 4:**
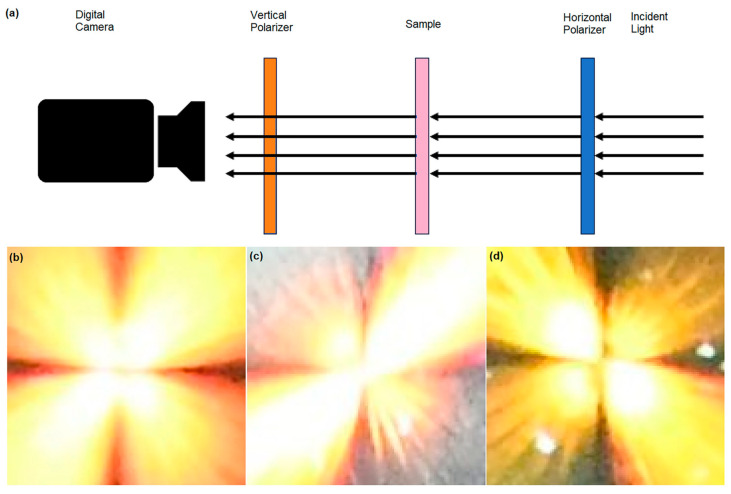
(**a**) Schematics of real-time imaging of plasmonic concentric circular gratings in between crossed polarizers. Real-time imaging of surface plasmon resonance transmitted from concentric circular gratings noted as (**b**) C2; (**c**) C3 and (**d**) C4 in between crossed polarizers excited by white light being normally incident on them.

To visually see the excitation of surface plasmon resonance on the concentric circular gratings, a digital camera (Nikon D850) was placed after the crossed polarizers, as illustrated in Figure 4a.

To avoid any image processing by the camera, the images were taken in manual mode focused on the thin films, and all the processing and light enhancement options were set to off. Figure 4b–d show surface plasmon resonance colors transmitted from concentric circular gratings C2, C3 and C4. As can be seen at first glance, from C2 (Figure 4b) to C4 (Figure 4d), the SPR color changes from a red tone to a more yellow tone, which is in agreement with the SPR peaks in Figure 3, which change from 628 nm to 612 nm and then to 598 nm. The other interesting phenomenon that can be seen in these photographs is the SPR butterfly effect, which is analogous to a Maltese cross phenomenon seen during the crossed polarized microscopy of some birefringent materials [32]. This occurs because the incident horizontally polarized light has two components, i.e., perpendicular and tangential to the radial lines at every point of the concentric circular pattern [19]. The perpendicular component excites SPR along the concentric circular grating, while the tangential component does not excite any SPR. Therefore, the transmitted light has a horizontal dumbbell shape [16] due to the radial polarization of the SPR propagating through the concentric circular grating. When the radially polarized dumbbell-shaped SPR light reaches the second polarizer, the light along the horizontal line becomes blocked. As a result, when a concentric circular grating is studied under crossed linear polarizers, only SPR from those grating areas which is not perpendicular to either polarizer is visible, thus forming a butterfly shape. Note that the white background light is being blocked fully in this configuration, and therefore the light that passes through is only that of the SPR. 

In their study, Wang et al. [18] presented polarized microscopy images featuring a Ag-coated concentric circular grating positioned between perpendicular linear polarizers. Their analysis revealed the intriguing phenomenon of an SPR butterfly effect, which can result from both radial and azimuthal polarizations of the excited SPR at the surface of concentric circular metallic gratings when placed between crossed linear polarizers. Notably, their simulation data indicated that above a plasmon resonant wavelength of 500 nm, radial polarization predominates, whereas below this threshold, azimuthal polarization becomes the dominant polarization state of SPR [18]. Consequently, the concentric circular gratings (C1–C5) in our study that excite SPR above 500 nm generate radially polarized surface plasmon resonance, and when positioned between crossed linear polarizers, they elicit a captivating butterfly effect observable in the transmitted light captured by the camera.

**Figure 5:**
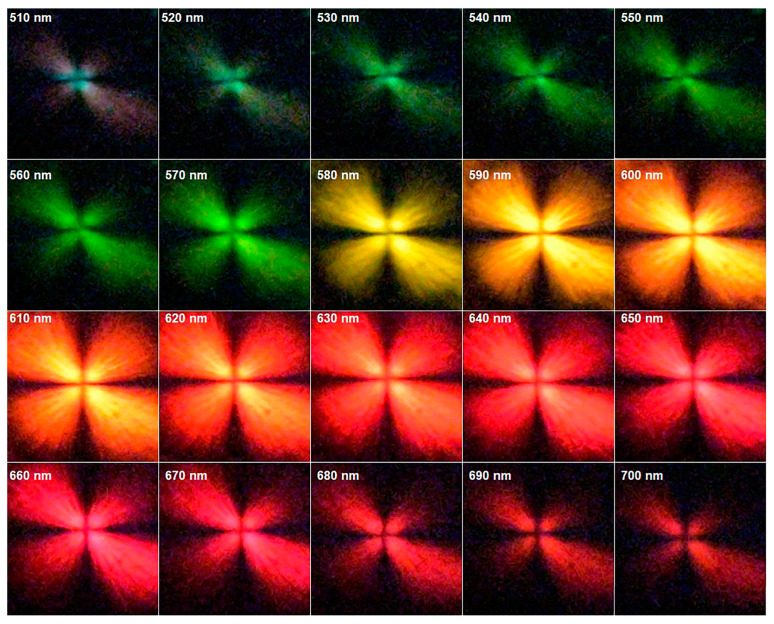
Real-time imaging of surface plasmon resonance from concentric circular grating (sample C2) in between crossed polarizers excited by various wavelengths of normally incident light.

Another feature in Figure 4 is that the images show the SPR visual wavelength band responses generated when polychromatic light is being shone on the entire crossed polarized setup. Therefore, the SPR photographs in Figure 4b–d are a mixture of different wavelengths of excited SPR light, from the wavelength at which a peak starts to the point at which it ends, as presented in Figure 3 in the form of plots. To improve this photographic method and the visual recognition of the excited SPR from the concentric circular gratings, a Kurios^®^ (Thorlabs, Newton, MA, USA) programmable liquid crystal tunable filter with narrow 10 nm bandpass filter was used in conjunction with the Halogen lamp to take real-time images between wavelengths of 510 nm and 700 nm at 10 nm intervals. The narrow output bandwidth light from the tunable filter was horizontally polarized and, as it was transmitted through the circular grating, it went through a secondary vertical polarizer before reaching the camera. In Figure 5, it can be seen that the intensity and color of the surface plasmon resonance signals from sample C2 change as the wavelength of the incident light is varied between 510 nm and 700 nm. It is visually clear that the maximum intensity falls between 580 nm and 630 nm, which corresponds to the C2 plot in Figure 3. A real-time video was recorded by the camera from the plasmonic response of C2 as the wavelength of incident light varied repeatedly from 510 nm to 700 nm with 10 nm bandwidth steps. The video showed a shimmering effect according to the intensity changes of the excited SPR within the adjusted range of wavelengths of the filter (See Supporting Information Video S1). 

**Figure 6:**
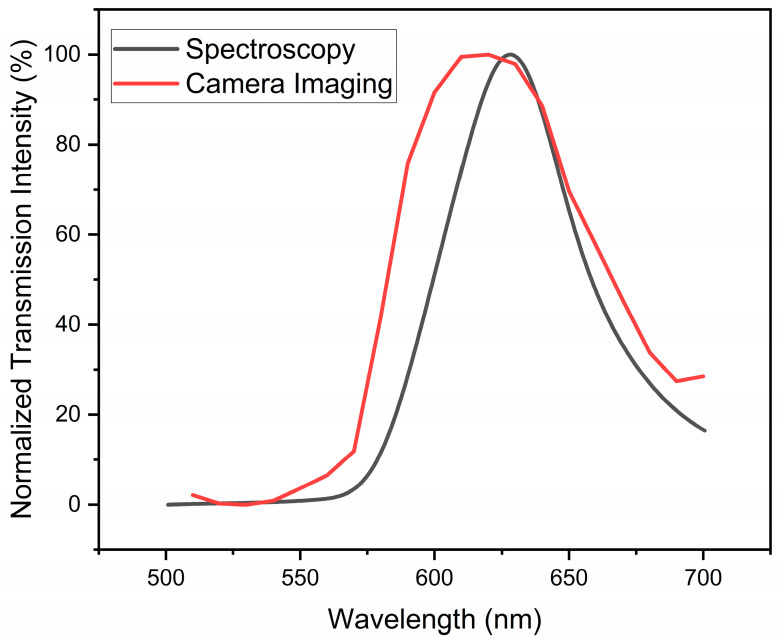
Camera- vs. spectrometer-obtained data for normalized transmission intensity of the surface plasmon resonance from the concentric circular grating C2 when placed in between crossed polarizers.

The intensity of each image in Figure 5 was measured using ImageJ v1.53k software and plotted against the wavelength of incident light. Figure 6 compares the normalized intensity of the transmitted SPR light from sample C2 when it was placed in between crossed polarizers measured by the spectrometer versus the real-time camera imaging technique. An obvious SPR peak can be seen in Figure 6, which is generated by the intensities of the camera images, and the peak is very similar to data collected by the spectrometer. To calibrate the camera, images of different wavelengths of light, obtained through the liquid crystal filter, which were tuned between 500 nm and 700 nm were taken, and their intensities were used as a reference to normalize the intensities extracted from the SPR images. In Figure 6, there is a slight shift in the position of the peak obtained by the camera (*λ*_max_ = 620 nm) compared to the spectrometric spectrum, which is due to the 10 nm bandwidth of the liquid crystal filter used in this study, which, as a result the intensities, were averaged within the 10 nm region. This study proves that the real-time imaging of SPR signals can be utilized as a high-accuracy colorimetric imaging technique instead of spectroscopic ones as a tool in various applications such as sensors or visual detections.

## 4. Conclusions

Using a combined lens–axicon interference lithography approach, concentric circular gratings with different pitch values were fabricated on thin films of azobenzene molecular glass (gDR1). The obtained concentric circular gratings were coated with gold, and their plasmonic behavior was studied using spectroscopic techniques. Alternatively, a real-time surface plasmon imaging technique using a digital camera, was introduced to study the excitation of surface plasmon resonance from the concentric circular gratings. The real-time surface plasmon imaging technique was in good agreement with the spectrometric results.

## Figures and Tables

**Table 1:** Pitch values and surface plasmon resonance maximum wavelengths of the concentric circular gratings fabricated in this work.

Sample ID	C1	C2	C3	C4	C5
Pitch value (nm)	660	584	574	553	469
1st SPR Peak Wavelength (nm) (marked by star)	730	628	612	598	545
2nd SPR Peak Wavelength (nm) (marked by dot)	1092	936	922	892	776

## Data Availability

Data underlying the results presented in this paper are not publicly available at this time but may be obtained from the authors upon reasonable request.

## Supplementary Materials

The following supporting information can be downloaded at https://www.mdpi.com/article/10.3390/mi14111981/s1, Video S1: Real-time video from plasmonic C2 in between crossed polarizers.
